# Intersectoral partnerships, a necessary path to overcome the challenges presented by Chagas disease

**DOI:** 10.3389/fpara.2023.1150041

**Published:** 2023-03-20

**Authors:** Marcelo Claudio Abril

**Affiliations:** Fundación Mundo Sano, Ciudad Autónoma de Buenos Aires, Argentina

**Keywords:** Chagas disease, mother-to-child transmission, public-private partnerships, cooperation, alliances

## 1 Introduction

American trypanosomiasis, known as Chagas Diseases (ChD), is a parasitic zoonosis caused by the protozoan *Trypanosoma cruzi*. Current worldwide estimates of infected individuals range between 6 and 7 million (Pan American Health Organization, 2023a). Historically, its main transmission route to humans was through infected feces or urine of triatomine insects (vector route).

Other transmission routes include oral (from consumption of contaminated food/drinks), transfusion or organ transplants from infected donors, and vertical (from an infected mother to her child during pregnancy or birth). Advances on control of vector transmission have granted preponderance to vertical or mother-to-child transmission (MCTC) as the prevailing route, causing the greatest number of new cases of ChD.

Originally, ChD was endemic in 21 countries of the Americas, mainly in rural and peri-urban areas. Nonetheless, the reality of ChD has changed due to migratory processes within and between countries. Today, most individuals with ChD are living in urban areas of countries with a history of vector transmission and there are numerous records of cases in migrant Latin American communities in other countries (i.e., Spain in Europe and the United Sates, among others).

Infection by *T. cruzi* causes chronic and irreversible cardiac, digestive, and neurological consequences in approximately 30% of those infected, contributing to a reduction in quality of life with serious effects that can lead to death.

Diagnostic techniques for the detection of *T. cruzi* are available, as well as drugs for its etiological treatment (benznidazole and nifurtmimox). These drugs are antiparasitic and have shown higher efficacy when administered during the acute phase or before 19 years of age. Nevertheless, most infected individuals are underdiagnosed and when infection is detected, access to treatment occurs late, i.e., when cardiac and/or digestive complications derived from the infection are already present or when women of reproductive age (WRA) have vertically transmitted the infection to their children.

After decades of efforts focused mainly on vector control, the gap between what is possible and what should be done, in terms of access to health for all those affected by ChD, represents a challenge that, to be achieved, requires the sum of capacities through the conformation of intersectoral partnerships. In these partnerships, different components of the health system coalesce, with coordinated efforts from governments as well as the participation of civil society organizations (CSOs) committed to this cause.

## 2 Horizontal cooperation background: The subregional initiatives

With the idea to boost and value partnerships between different actors, it´s important to highlight the horizontal technical cooperation between countries spearheaded by the Pan American Health Organization (PAHO)/World Health Organization (WHO) since the early 90´s (Pan American Health Organization, 2023b). This was implemented through the creation of four Subregional Initiatives for the Prevention and Control of ChD (Southern Cone, Central America and Mexico, Andean Countries and Amazonian Countries).

Important advances were achieved, such as the reduction in the number of acute cases and in the intra-domiciliary infestation levels of vector insects, leading to the certification of interruption of vector transmission in 17 countries. Moreover, the implementation of universal screening of blood donors was achieved in 21 endemic countries.

Despite these important milestones, there is an estimated 30,000 new cases each year, approximately 12,000 annual deaths and around 8,600 births of children infected through MTCT in the Americas (Pan American Health Organization, 2023a).

Other important cooperation actions for ChD in the Americas include the Japanese International Cooperation Agency (JICA) with projects in Central America since 2000 (Japan International Cooperation Agency (JICA), 2023), the work of Doctors without Borders from 1999 to 2016 in collaboration with the health ministries of different countries, and the conformation of the Chagas Platform by Fundación Ciencia y Estudios Aplicados para el Desarrollo en Salud y Medio Ambiente (CEADES) from Bolivia and the Barcelona Institute for Global health (ISGlobal) from Spain (ISGlobal, 2023), providing healthcare for adult ChD patients through a network of centers throughout Bolivia.

## 3 ChD in the current context of the neglected tropical diseases and the global agenda

Through the work of different academic scientists in collaboration with the WHO, the term NTDs started being mentioned in 2005, to group different diseases which affect mostly vulnerable populations living in tropical and sub-tropical areas of the world. ChD was included in 2006 (Molyneux et al., 2021) and the list is currently comprised of 20 diseases or conditions.

In 2010, the WHO elaborated the first NTD roadmap with objectives for each of the diseases to be met by 2020 (World Health Organization, 2012). Despite important advances, many of these goals were unmet. The experience obtained during 2010 – 2020 taught us that greater multisectoral action was needed.

In 2015, the United Nations (UN) approved 17 objectives as part of the 2030 Agenda for Sustainable Development. The Sustainable Development Goals (SDGs) constitute a universal call for action to end poverty, protect the planet and improve the lives and prospects of people around the world (United Nations et al., 2023). Within these goals, SDG 3 is related to health and wellbeing, specifically mentioning the elimination of NTDs, and SDG 17, which is transversal to the previous 16 goals in acknowledging the need to have partnerships between governments, the private sector and civil society.

In 2021, the WHO presented a second roadmap for the NTDs, elaborated through a consultative process including different actors, with objectives and goals to be met by member states by 2030 (World Health Organization, 2020). Five of these objectives relate to ChD, including the verification of the interruption of intra-domiciliary vector transmission, as well as transfusion and transplant transmission; elimination of congenital ChD; and the goal of reaching 75% antiparasitic treatment coverage in at risk population.

Main changes captured in this roadmap consist of shifting from a specific focus on each disease to an integrated focus comprising all NTDs, aiming to guarantee the commitment and leadership of the countries, working closely with other partners, including CSOs. This roadmap places people and communities in the center of the efforts to improve their health and wellbeing.

Therefore, the SDGs and the goals set by the new roadmap for NTDs are intimately tied and dependent on each other. Moreover, given that the SDGs themselves are interdependent, the WHO highlights the importance of conforming solid partnerships to be able to meet the goals set by both the UN and the WHO Roadmap and the advantages of public-private associations to significantly facilitate progress, based on previous experiences.

Presently achieved milestones are a testament of the support and dedication of those involved in the fight against NTDs since their first mention in 2005, followed by the commitments assumed through the London Declaration on NTDs in 2012 (Uniting to Combat Neglected Tropical Diseases, 2023) and the meeting held by WHO in 2017 with all its partners. These advances are evidence of the immense potential of collaborative work, guaranteeing that NTDs remain in a prominent position within the global health agenda.

### 3.1 World Chagas disease day – the role of patient associations

ChD has been called a “silent and silenced” disease, due to its slow clinical progression and its pervasiveness within vulnerable populations “without a voice”. Thanks to an initiative of the International Federation of Associations of People Affected by Chagas Disease (FINDECHAGAS) (FINDECHAGAS, 2023), in 2019, the 72nd World Health Assembly (WHA) approved World Chagas Disease Day as April 14 (World Health Organization, 2023) with the goal of raising awareness.

FINDECHAGAS was founded in 2010 as a CSO comprised of affected individuals, family members, friends, and professionals with the goal to strengthen collective action towards ChD.

Thus, World Chagas Disease Day is the best and more complete example of the role that a CSO, especially a patient association, can have in the promotion, control, or elimination of a disease, when they are given a voice.

### 3.2 Uniting efforts and capacities – the role of CSOs and international forums

Different CSO´s have come together to support and perform research to improve diagnosis and treatment of ChD and to implement projects related to all the transmission routes present in endemic and non-endemic countries.

These efforts to increase awareness of ChD have proven to be more efficient through the collaboration between organizations that are aligned towards the same goals: gain visibility for ChD compatible with its burden, guarantee the necessary resources to overcome current access barriers, foster the demand from civil society and increase the provision of care from health professionals.

This is the case of the alliance Uniting to Combat (UTC) NTDs, born from the 2012 London Declaration for NTDs with the vision to support the WHO Roadmap through public-private associations and collaborative work with the WHO and partners across different sectors. As a member of this alliance, Fundación Mundo Sano (FMS) (Mundo Sano, 2023), the only Latin American member, successfully proposed the inclusion of ChD within the original list of diseases to be supported through this international initiative. FMS is a CSO that has been working to prevent transmission of ChD and promote D&T since 1993.

Another example is the Global Chagas Coalition (2023), an open collaborative alliance that was initially founded by FMS, Drugs for Neglected Diseases Initiative (DNDi) (DNDi, 2023), the Sabin Vaccine Institute (SVI) (Sabin Vaccine Institute, 2023), CEADES (2023), ISGlobal, and FINDECHAGAS.

It is exciting to see how the conformation of partnerships and consortiums have been multiplying. In 2018, FMS, DNDi and the International Development Research Center (IDRC), under the leadership of the Ministry of Public Health and Social Welfare and the collaboration of San Carlos University of Guatemala, the Association for Research and Social Studies and PAHO/WHO, are implementing the Project Alliances for the Elimination of ChD as a public health problem in Central America and Mexico (IDRC/CRDI, 2023).

In 2021, the consortium ChagasLAMP, financed by the Global Health Innovative Technology Fund (GHIT) of Japan and FMS (GHIT Fund, 2023), was created to validate a new diagnosis technique for ChD in newborns and evaluate the performance of rapid tests in chronic patients in Bolivia, Paraguay, and Argentina.

Additionally, through the provision of funds by UNITAID, the project “Communities United for Innovation, Development and Care of Chagas Disease (CUIDA Chagas)” (UNITAID, 2023), led by the National Infectious Disease Institute “Evandro Chagas” of the Oswaldo Cruz Foundation (FIOCRUZ) of Brazil is being implemented.

### 3.3 Conformation of an Ibero-American initiative: “Not a single baby with Chagas disease”

Currently, both in and outside of Latin America, MTCT is the main route of transmission of ChD. This new epidemiological reality drove FMS to appeal to the awareness of this transmission route through an innovative communication strategy with the aim to engage, sensibilize, and gain commitment from society towards this issue.

The campaign “Not a single baby with Chagas Disease” was launched by FMS in 2019, through the support of an audiovisual spot called “Heritage,” which breaks the traditional paradigm of communication on ChD. During many years, communication on ChD was plagued with stereotypes and social representations that only reflected the burden of the disease without showing the real possibility of its treatment. The spot itself, as well as all the elements of the communication campaign, propose that it is possible to attain the goal of no babies born with ChD by 2030, using positive language and in alignment with WHO goals. This goal can be met by making sure the conditions are given so that all babies born infected with *T. cruzi* are treated and all WRA have access to D&T to eliminate the risk of MTCT.

The campaign was formally launched in the headquarters for the Ibero-American General Secretariat (SEGIB) in Madrid (Spain) and the Secretary-General of the organization at that time, Rebeca Grynspan, urged FMS to work together with Ibero-American countries to create an initiative or potential program at the regional level (Secretaría General Iberoamericana, 2023).

Subsequently, Argentina formally presented the proposal for such an initiative and Brazil quickly adhered, followed by Colombia and Spain.

The Ibero-American initiative “Not a single baby with Chagas: the path towards new generations free of Chagas Disease” was officially announced during the XXIII Ibero-American Summit of Heads of State and Government in 2021, where the member countries considered appropriate to give priority attention to this disease, one of the most important and prevalent NTD in the Americas, setting the goal to eliminate MTCT in member countries.

To reach this goal, it is essential to consolidate a series or intermediate steps: guarantee access to D&T of pregnant women, WRA, teenagers and girls, as well as to consolidate the results obtained in the control of vector transmission given that if preventive measures are not sustained, the transmission cycles will inevitably restart.

In 2022, four guest members, Paraguay, Guatemala, Honduras, and El Salvador, adhered to support the initiative. Additionally, WHO, PAHO, ISGlobal, DNDi, and the Chagas Coalition fully support this initiative while FMS acts as the Technical Unit.

The path travelled is a testament to the importance of continuing to work together with different sectors, at all levels, to keep adding partners and to effectively communicate the problem to make not only ChD more visible, but also the rest of the NTDs.

## 4 Concluding remarks

The history of ChD and the efforts provided for its control and elimination is long and has gone through different stages. Vector control advances currently present a scenario were the attention of those affected are a priority. The coordination and sum of capacities of all and each one of the committed partners - from PAHO/WHO, to the national health systems, the CSOs and patient associations - is necessary to guarantee access to health in an equitable manner.

Some of the experiences mentioned above allow us to be optimistic; they evidence the willingness and conviction of the different sectors to work collaboratively and through horizontal cooperation.

The current challenge is to ensure that different existing initiatives and consortiums, and those that might arise in the future, establish communication channels to avoid the duplication of efforts, to be able to obtain better results based on the available resources, and to provide a definitive solution for ChD in its multiple dimensions.

## Author contributions

The author confirms being the sole contributor of this work and has approved it for publication.
